# Tuning biological processes *via* co-solutes: from single proteins to protein condensates – the case of α-elastin condensation†

**DOI:** 10.1039/d4sc07335e

**Published:** 2025-02-24

**Authors:** B. König, S. Pezzotti, G. Schwaab, M. Havenith

**Affiliations:** a Lehrstuhl für Physikalische Chemie II, Ruhr-Universität Bochum Bochum 44780 Germany martina.havenith@rub.de

## Abstract

Protein condensates as membrane-less compartments play a pivotal role in cellular processes. The stabilization of protein condensation can be tuned using cosolutes which directly impact biological function. In this study, we report the result of a rigorous study of the influence of cosolutes changes on hydration entropy and enthalpy upon condensate formation, by means of THz-calorimetry. Our results unveil quantitative insights into the fine tuning of the free energy imbalance, *via* hydrophobic/entropic and hydrophilic/enthalpic hydration which can result in cosolute-mediated stabilization or destabilization of protein condensates. These results shed new light on the regulatory potential of co-solutes within cells, to tune Liquid–Liquid Phase Separation (LLPS). Furthermore, we demonstrate the transferability of the underlying molecular concepts of cosolute addition to two fundamental biological processes: protein folding and denaturation. This study provides a blueprint for controlled modulating LLPS *via* cosolute additions, with promising implications in both biological and medical applications.

## Abstract 2

THz-calorimetry reveals changes in hydration entropy and enthalpy upon liquid–liquid phase separation. Our results are essential to predict how variations in the cosolute concentration can regulate biological functions.

## Graphical abstract

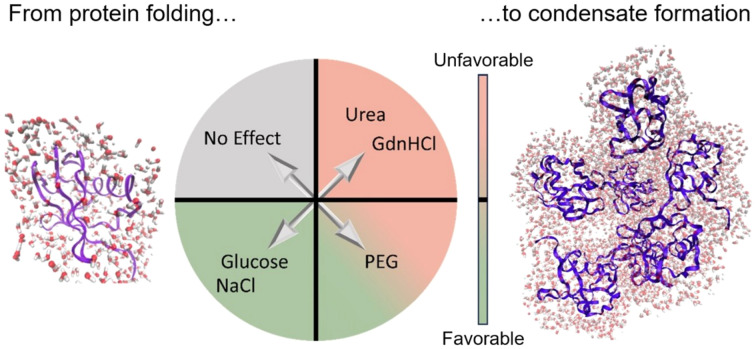

## Introduction

Cosolute effects on protein stability have been extensively studied in the context of protein folding and denaturation in terms of both a direct interaction with proteins and a local change in protein solvation.^1–9^ Cosolutes are commonly categorized as osmolytes, denaturants, and crowders, each with a specific influence on protein thermodynamics. The well-established picture is the following: osmolytes (*e.g.* NaCl and glucose)^1,2,6,10–13^ stabilize proteins by preferential hydration, denaturants (*e.g.* urea and guanidinium hydrochloride (GdnHCl))^1,6,14–18^ destabilize *via* hydrophobic interactions, and crowders (*e.g.* polyethylene glycol (PEG))^1,6,19–21^ impact thermodynamics *via* steric effects, which can promote either protein–protein or protein–solvent interactions.^1^ However, these concepts for a single protein cannot be transferred directly to understand the influence of cosolutes on the formation of biomolecular condensates.^22^

Biomolecular condensates that are reversibly formed upon LLPS play a pivotal role in cellular processes, by serving as *e.g.* dynamic regulators, as well as local hot-spots for reactions and the formation of neurotoxic aggregates.^23–27^ The crowded cellular environments contain a plethora of organic and inorganic cosolutes.^25–32^ Changes in the cosolute concentration within cells act as switches for LLPS.^33^ This has medical implications, as understanding cosolute-driven regulation could lead to novel treatments for LLPS-related diseases such as neurodegenerative disorders.^34,35^ However, the molecular mechanisms by which cosolutes impact LLPS, for instance by altering biomolecule hydration properties, remain largely unexplored and are yet a challenge for both theory and experiment.

In the previous paper on α-elastin, we used THz spectroscopy to quantify local hydration enthalpy and entropy changes upon LLPS of α-elastin in real time, as deduced directly from experimental THz spectroscopy data.^36^ The central hypothesis at the heart of our “THz-calorimetry” method is that variations in local solvation motifs dictate changes in solvation free energy. We tested this hypothesis for alcohols and glycerol mixtures and could verify a linear correlation between spectroscopic observables in the difference extinction spectrum of the solvated solute compared to a reference sample and the changes in the limiting partial molar excess entropy and enthalpy, as measured before with well-established calorimetric techniques. As a consequence, changes in solvation thermodynamics are then correlated with changes in the experimentally observed THz spectra, associated with changes in the local solvation motifs. In further studies, the same concepts could be transferred to study LLPS of FUS and alpha-elastin.^36,37^ As a result we proposed that hydrophobic solvation dominates the entropic solvation term, while hydrophilic solvation mainly contributes to the enthalpy. Both terms were found to be in the order of 100 s of kJ mol^−1^ for α-elastin, which is more than one order of magnitude larger than the total free energy changes at play during LLPS, but almost compensates.

Enthalpy – entropy compensation is a well-discussed topic in biology.^38,39^ However, a small entropy/enthalpy imbalance is sufficient to initiate LLPS which can be tuned by small changes in temperature, concentration, and protein hydrophilicity. In the present paper we use the same method to probe the impact of adding cosolutes, which are well known to tune the imbalance and thus drive LLPS.

Here, we report the results of THz calorimetry, which rely on the hypothesis that changes in local hydration motifs can be probed using changes in the THz spectra to understand how osmolytes impact the hydration of proteins in the process of LLPS. Here we present new data unraveling imbalances in free energy upon the addition of cosolutes. This is of biological relevance, since cosolutes serve as switches for LLPS within a cell. Here, we focus on the LLPS behavior of α-elastin, which serves as an excellent prototype for studying the LLPS of intrinsically disordered proteins.^40^

## Materials and methods

### Sample preparation

α-Elastin was purchased from Elastin Products Company Inc. (EPC, Owensville, Missouri). Dried α-elastin was dissolved in phosphate buffer, 1× PBS (137 mM NaCl, 2.7 mM KCl, 10 mM Na_2_HPO_4_, 1.8 mM KH_2_PO_4_, ultrapure water, pH = 7.4).^41^ 20 mg mL^−1^ α-elastin was solvated in 1× PBS and the following cosolutes were added up to the final concentration: 0–1.2 M NaCl, 0–0.5 M GdnHCl (99.5%), 0–0.5 M urea (99.5%), 0–15% (w/v) glucose (d-(+)-glucose, 99.5%), and 2–10% (w/v) 20 kDA PEG.

### FTIR measurements and data analysis

Low frequency (THz) spectra were recorded with a Fourier-transform infrared (FTIR) spectrometer (Vertex 80v; Bruker, Billerica, MA) using a mercury vapor lamp as a radiation source and a helium-cooled silicon bolometer (Infrared Laboratories, Tucson, AZ) as a detector in the spectral range of 60–650 cm^−1^. The FTIR sample compartment was equipped with a single reflection attenuated total reflection (ATR) unit (MVP-Pro; Harrick Scientific, Pleasantville, NY) with a temperature-controlled 500 μm diameter diamond crystal (Harrick Scientific). The FTIR interferometer compartment was evacuated (approx. 3 mbar) and the sample compartment was constantly purged with nitrogen (approx. 1 bar) to minimize the absorption by water vapor. Spectra were collected in intervals of 2 min, each with an average of 64 scans and a spectral resolution of 2 cm^−1^ until equilibrium has been reached. After each measurement, the diamond crystal was cleaned using ultrapure water, 0.5 M NaOH solution (Sigma Aldrich), and isopropanol (Sigma Aldrich).

ATR absorption spectra *α*(*ν*) were calculated using eqn (1):1
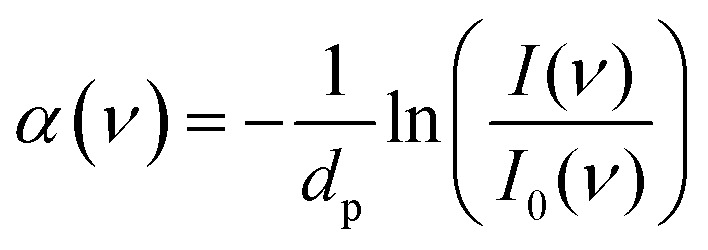
where *I*(*ν*) and *I*_0_(*ν*) are the frequency-dependent intensities of the sample and reference, in which the cleaned diamond surface served as the reference. In the case of strongly absorbing samples, such as aqueous solutions, the decay of the electric field at the interface is no longer purely real and the penetration depth, *d*_p_, represents an upper limit for the propagation of the evanescent wave into the sample:^42^2
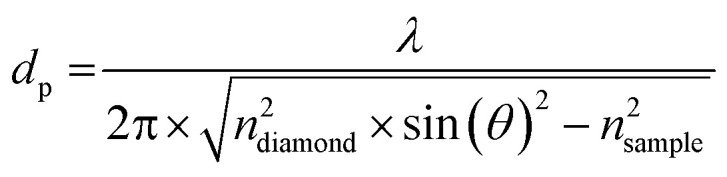


The incident angle was *θ* = 45°. The refractive index of the diamond is *n*_diamond_ = 2.38. For the sample we assumed the same refractive index as that of water (*n*_sample_ = 1.5) independent of the frequency. The high protein density inside LLPS droplets could affect the refractive index of the sample relative to the dilute phase and therefore the resulting penetration depth. However, because many previous studies showed that the condensed phase retains a large fraction of water.^43,44^ The refractive index of the sample is assumed to be equal to that of an aqueous solution.

Difference absorption spectra (Δ*α*) were deduced by subtracting the initial spectrum of the diluted protein (serving as the reference) from the subsequent recorded spectra.

3 Δ*α*_n_(ν) = *α*_n_(ν) − *α*_1_(ν)

After 60 minutes, equilibrium was reached and no further changes in respect to formation of a protein condensate were observed.

### Principles of THz calorimetry

Qualitative experimental estimation of solvation free energies is often done *via* surface area or hydropathy scale models. However, as summarized in a review by Rego *et al.*,^45^ these models are limited to simple cases due to the context-dependent nature of hydrophobicity. As pointed out in many previous simulation studies,^46–54^ different factors come into play: (i) the hydration of hydrophobic surfaces or patches depends on local morphological characteristics, such as surface curvature; (ii) the hydrophobicity of a residue in a protein depends very much on the local surrounding.^55^ Therefore, simple additive models, based on the number of hydrophobic and hydrophilic groups, are not sufficient.

Traditional calorimetry can provide thermodynamic properties in a macroscopic sample under equilibrium conditions. Alternatively the solvation free energy is estimated based on the known number of hydrophobic and hydrophilic groups only. However, as pointed out in previous publications, it is not just the number of hydrophobic groups which counts, but also surface chemical patterning influences hydrophobicity, see Rego *et al.*^56^ In these studies it could be shown that chemical patterns with a fixed polar content can be designed that vary widely in their hydrophobicity, as quantified using the free energy cost Δ*G*_cavity_ of creating a cavity next to the patch. For patches with the same polar content, clustering the polar groups enhances hydrophobicity. We have developed a new approach based on spectroscopic observables of local solvation motifs, going beyond restrictions of traditional calorimetry approaches.

In previous studies we could show that limiting partial molar excess entropy and enthalpy changes are linearly correlated with changes in the THz molar extinction spectrum of the solvated solute compared to the infinitely diluted bulk water spectrum. These differences in the THz spectra provide information on the change in intermolecular interactions between the solute and its hydration water molecules. The intermolecular modes which are probed are the intermolecular stretching of the H-bonds formed between water molecules (100–300 cm^−1^) and the librational, *i.e.* the hindered rotations of water molecules within the H-bond network (300–700 cm^−1^). These modes, H-bond stretch and librational mode are especially sensitive to the radial and the angular part of the intermolecular potential energy surface, respectively. Any changes in the molar extinction spectra are related to changes in local solvation motifs, as could be shown by a joint experimental and simulation study.^55^

In general, the total solvation free energy is obtained by summing the Gibbs free energies of these two steps:

4 Δ*G*_solv_ = Δ*G*_cavity_ + Δ*G*_insert_ = Δ*H*_cavity_ − *T*Δ*S*_cavity_ + Δ*H*_insert_ − *T*Δ*S*_insert_

where Δ*H*_cavity_, −*T*Δ*S*_cavity_, Δ*H*_insert_, and −*T*Δ*S*_insert_ are the partial contributions to the free energy (Δ*G* = Δ*H* − *T*Δ*S*).

Δ*G*_cavity_ quantifies the volume exclusion effect and the associated perturbation on the surrounding water network wrapped around the solute, while any attractive intermolecular interaction, *e.g.*, van der Waals, electrostatic and H-bonding, is included in Δ*G*_insert_. Entropic and enthalpic terms due to pure water–water interactions will cancel and thus do not contribute to the solvation thermodynamics. This formalism is consistent with standard thermodynamic concepts in the previous studies of Ben-Amotz and Underwood,^57^ where Δ*G* = *E*_UV_ − *TS*_UV_, with *E*_UV_ and *S*_UV_ being solute–solvent interaction energy and entropy, respectively, while the solvent–solvent terms *E*_VV_ and −*TS*_VV_ cancel out, *e.g. E*_VV_ − *TS*_VV_ = 0. For small solutes, such as alcohols, −*TS*_UV_ is dominated by the cavity formation process (−*T*Δ*S*_cavity_), as shown by theory^58^ and experiments,^59^ while attractive solute–water interactions (solute insertion step) mostly contribute as an additional enthalpic term, *i.e.* to *E*_UV_.

The underlying hypothesis of THz calorimetry is the following: if we can experimentally probe the change in two distinct water populations, representative of changes in cavity formation around a hydrophobic patch or hydrogen bonding to a hydrophobic group, with the changes accounting for both the number and quality of the hydrogen bonds surrounding the solute, then we can experimentally deduce the changes in solvation free energy from spectroscopic observables.

Therefore, we investigated the low frequency spectra of the solvated prototype solutes compared to bulk water in an endeavour to deduce spectroscopic observables for the local solvation motifs. More specifically, we looked for a correlation between the change in the specific spectroscopic observable and the corresponding limiting excess molar thermodynamic functions. Furthermore, we assume that the absorption of the intramolecular modes of the solute itself in the respective frequency range (100–600 cm^−1^) can be neglected and the spectrum is dominated by water absorption.

In a joint experimental and simulation study, we could show that any positive change in amplitude in the molar extinction spectrum at around 150–165 cm^−1^ is indicative of an increase in intermolecular hydrogen bonds which are weaker compared to those in bulk water (where the maximum absorption is around 195 cm^−1^).^55^ The red-shifted mode could be assigned to the collective mode of hydrogen bonds with a decreased tetrahedrality as expected when a cavity is formed (or “wrapped”) associated with a hydrophobic solvation mechanism. Indeed, these H-bonds formed between hydration water molecules around a hydrophobic group or patch are spectroscopically, structurally, and dynamically different from the H-bonds in bulk liquid water. These H-bonds have a decreased tetrahedrality and lower partial local entropy than in bulk water (as confirmed by DFT-MD and classical MD simulation studies).^60^ The more the water network is perturbed to accommodate the solute, the more water molecules contribute to this specific band which is red-shifted from bulk water.

On the other hand, the fingerprint of hydration water bound to a hydrophilic group lies in the frequency range of the libration (300–700 cm^−1^). In bulk water, this band is inhomogeneously broadened: soft librations are contributing at lower frequencies <400 cm^−1^, and hard or stiffer librations at higher frequencies 400–600 cm^−1^, indicative of less or more hindered orientational motions of water molecules, respectively. The characteristic bound population signature in this spectral region originates from steric constraints in water rotational motions induced by the proximity to and direct H-bonding with the solutes.^61–63^ This causes a decrease in the partial amplitude attributed to soft librations (400 cm^−1^) and an increase at hard or stiff librations (600 cm^−1^) with respect to bulk. This results in a negative Δ*α* around <400 cm^−1^ when taking the difference between the sample and the bulk water spectra and a positive Δ*α* between 400 and 600 cm^−1^. As a consequence, the difference THz spectra show a characteristic, linear intensity increase in the >400 cm^−1^ range. The extent can be quantified using 
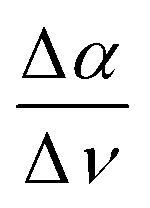
 obtained from the linear fitting of the difference amplitude in the 400–600 cm^−1^ range.^64^ The slope 
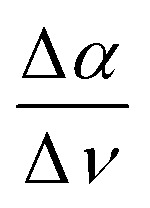
 is a measure of the attractive solute–hydration water interactions that stabilize solvation with a favorable enthalpic contribution: the more bound water molecules and the stronger they interact with the proteins, the more constrained the hydration water orientational dynamics, and the larger 
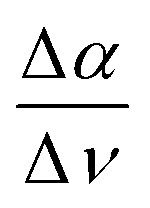
 in the frequency range between 400 and 600 cm^−1^. Therefore, the two spectroscopic observables for the cavity-wrap (indicative of hydrophobic hydration) and bound water (indicative of hydrophilic groups) are generic markers for hydrophobic and hydrophilic solvation.^64–66^

More precisely, we directly quantify changes in hydration free energy changes using:

5 Δ*S*_wrap_(conc, *T*) = Δ*α*_wrap_(conc, *T*)Δ*S̄*_wrap_, Δ*S̄*_wrap_ = −4.4 J mol^−1^ K^−1^ cm

6
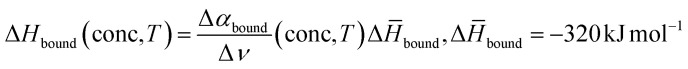
where Δ*α*_wrap_ and 
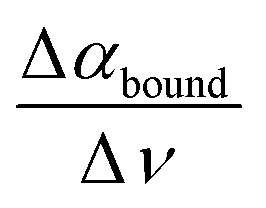
 are spectroscopic observables. In a previous study on solvated alcohols, we could show that the amplitude of the difference extinction spectrum at the peak around 150–165 cm^−1^, denoted as Δ*α*_wrap_, is proportional to the difference in the limiting molar excess mixing entropy for this alcohol and temperature compared to the same alcohol at a fixed value for a given reference temperature.^64^ Spectroscopic data recorded for four different alcohols at five temperatures were used to fit the scaling factor Δ*S̄*_wrap_ = −4.4 J mol^−1^ K^−1^ cm for all alcohols and temperatures. We used the well-known data for the limiting solvation entropy of alcohols (as measured by standard calorimetry) to fit the correlation factor Δ*S̄*_wrap_, because their limiting excess mixing entropy is dominated by the cavity formation process, as predicted by theory,^58^ as well as found in previous experimental studies.^59^ The partial contribution of hydrophobic hydration water is much more temperature dependent as the hydration water is bound to the polar group. Thus, the temperature dependent changes in free energy for these alcohols were governed by the temperature dependent changes of the cavity wrap water population.

In a later joint experimental and simulation study, we could attribute the librational feature, consisting of a decrease in molar extinction around 300 cm^−1^ and an increase between 400 and 600 cm^−1^ compared to bulk water, to water hydrogen bonded to the solute. This characteristic observable, as quantified using the slope or derivative of the difference between 400 and 600 cm^−1^, *i.e.* slope 
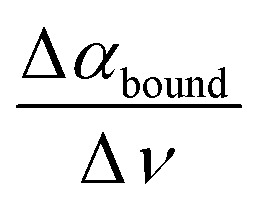
, is well suited to probe the bound water, which is mostly sterically hindered compared to the bulk and thus has a major impact on the librational mode.

We chose glycerol–water mixtures as the candidate, which yielded a linear correlation between the spectroscopic observable (the slope) and the changes in excess mixing enthalpy – again against the infinitely diluted limit.^64,67^ The scaling factor between the characteristic observable 
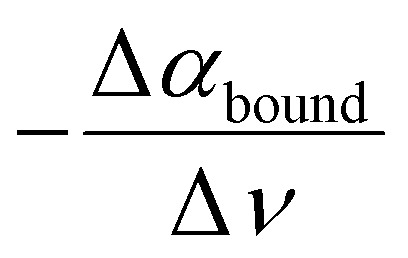
 and the molar excess mixing enthalpy could be deduced from a fit to Δ*H̄*_bound_ = −320 kJ mol^−1^.^68^

Further applications of THz calorimetry include alcohols, DMSO, glycerol and proteins undergoing LLPS.^59,64,65,68–71^ These studies supported the hypothesis that variations in local solvation environments dictate relative changes in solvation free energies that are well quantified by changes in the spectroscopic observable (which were found sensitive to variations in temperature, concentration, local surface pattern and morphology, *etc.*), which is the central hypothesis at the heart of THz-calorimetry.

## Results and discussion

The previously introduced ATR sedimentation assay^37^ is used to study the effect of cosolute protein hydration while protein condensates are formed and sink to the bottom of the ATR cell. Experiments are carried out at two temperatures of 20 & 32 °C or 32 & 40 °C. We investigated the impact of cosolutes using a prototype cosolute for each of the three classes crucial for protein stabilization: the osmolyte (NaCl and glucose), denaturant (GdnHCl and urea), and crowder (PEG).

**Fig. 1:**
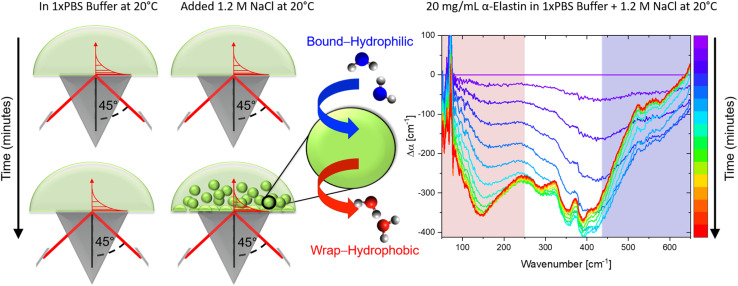
ATR sedimentation assay for measuring THz spectra upon LLPS. (Left) Schematics illustrating the formation of protein condensates upon LLPS and their sedimentation on the ATR crystal during the measurements. (Right) The measured absorption (Δ*α*) as a function of time (for one example), plotted as a difference with respect to the diluted protein phase (initial spectrum). During the time-series, the sedimentation of protein condensates causes the observed changes in the THz features associated with cavity-wrap (hydrophobic, red) and bound water (hydrophilic, blue) hydration contributions, as discussed in the text.

**Fig. 2:**
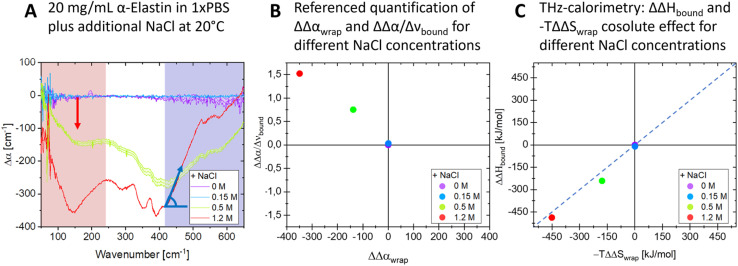
The addition of NaCl affects the release of cavity-wrap hydration water and the retention of hydrogen bound water. (A) Difference THz spectra upon LLPS as a function of increasing cosolute concentrations, shown for NaCl. The changes in cavity-wrap and bound water features are highlighted in red and blue, respectively. These changes are quantified using Δ*α*_wrap_ and 
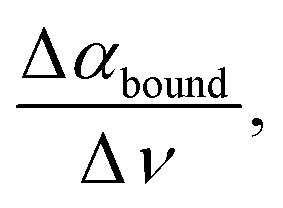
 shown in the plot and defined in the text. (B) The plot shows ΔΔ*α*_wrap_ and 
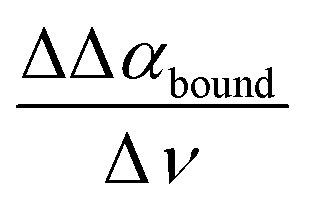
 for three different cosolute concentrations. (C) Changes in ΔΔ*H*_bound_ and −*T*ΔΔ*S*_wrap_ solvation upon the addition of NaCl. ΔΔ*H*_bound_ and −*T*ΔΔ*S*_wrap_ are the partial contributions to hydrophilic and hydrophobic hydration as deduced by means of THz-calorimetry, see methods and eqn (5) and (6).

In Fig. 1 we display a time series of THz spectra for a 1.2 M buffer solution and 20 mg mL^−1^ α-elastin at 20 °C. Time zero corresponds to the diluted protein phase (prior to LLPS), and with increasing time LLPS droplets form and sink to the bottom of the ATR unit, leading to changes in absorption as probed using the evanescent wave. Fig. 2A displays the difference spectra between the last (at 60 minutes) and the initial measurement (see eqn (3)) for increasing concentrations of sodium chloride (NaCl). At 0 M NaCl, α-elastin does not undergo LLPS (the critical transition temperature for protein condensation is 24 °C). As a consequence, the diluted phase persists over time, resulting in a zero line for the difference THz spectrum. With increasing NaCl concentration, protein condensates are formed even below 24 °C (Fig. S1 ESI †). Here, we observe the two characteristic spectroscopic features of LLPS in the difference spectra.^36,37,66,69^ In a nutshell, the first one, highlighted in red and referred to as cavity-wrap spectroscopic population, is centered around 150 cm^−1^. The second observable 
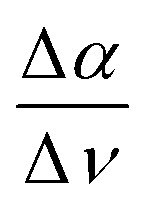
 refers to the minimum in the absorption around 300 cm^−1^ with an increasing absorption for higher frequencies (highlighted by blue in the figure). As explained in the Method section, these spectroscopic observables are a measure of the attractive solute–hydration water interactions between the hydration water and hydrophilic groups that stabilize solvation with a favorable enthalpic contribution: the more bound water molecules and the stronger they interact with the proteins, the more constrained the hydration water orientational dynamics, and the larger the slope.

Previously, we combined atomistic molecular dynamics (MD) simulations and terahertz (THz) spectroscopy to determine the solvent entropy contribution to the formation of condensates of the human eye lens protein d-crystallin.^72^ The MD simulations revealed an entropy tug-of-war between water molecules that are released from the protein droplets and the ones that are retained within the condensates. These two categories of water molecules could also be assigned to spectroscopically observed changes in the THz spectrum upon protein condensation. We compared the experimentally derived changes in these two water populations and the computationally determined changes based on the same approach as that used in the present paper. The two spectral features associated with the involvement of two distinct categories of hydration water in the LLPS process were denoted as “cavity-wrap” and “bound”. These spectral features could be mapped to the released and retained water molecules, respectively, as defined *via* MD simulations. The entropic tug-of-war between these two classes of hydration water was shown to play a crucial role in the process of LLPS, whereby the released water molecules gain entropy and the retained waters pay an entropy penalty due to increased confinement in the dense condensate phase. We could disclose that both partial hydration water contributions change upon LLPS: cavity-wrap water is released from the protein surface upon condensate formation, while water bound to hydrophilic groups is retained as much as possible, keeping the protein condensate in the liquid state.^32,73^ For LLPS the cavity-wrap contribution dominates changes in solvation entropy. On top of the cavity-wrap contribution, the hydration of polar groups interacting with bound water molecules also involves an additional enthalpic term due to the strong solute–water interactions at play. This term dominates the change in solvation enthalpy upon LLPS. Therefore, to understand how hydration water drives LLPS and how these driving forces are affected by co-solute addition, it is sufficient to consider the entropic contribution from the wrapped water and the enthalpic contribution from bound water. Further details can be found in ref. 64, 66 and 68.

In the present study, we wanted to use THz calorimetry to quantify the impact of cosolutes on the change in solvation. Thus, Δ*α*_wrap_ and 
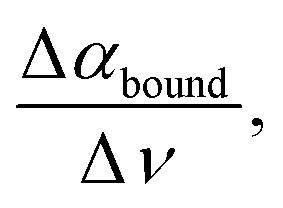
 denote in the following the changes in amplitude and slope upon the addition of the respective cosolute. As can be seen in Fig. 2A, both spectroscopic observables are affected. As stated in our introduction, we use THz spectroscopy to quantify local hydration enthalpy and entropy changes upon LLPS.^36^ The central underlying hypothesis is that the observed variations in local solvation motifs dictate changes in solvation free energy. We tested this hypothesis for alcohols and glycerol mixtures. We applied the same method in our previous studies on α-elastin^36^ and human eye lens protein d-crystallin^72^ and found a good agreement between the predicted and the deduced thermodynamic quantities. Based upon Δ*α*_wrap_ and 
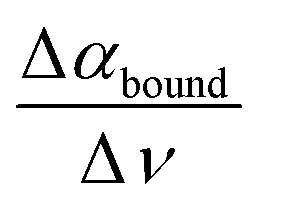
 we can deduce Δ*S*_wrap_ and Δ*H*_bound_, *i.e.* the estimated change in solvation entropy and enthalpy upon the addition of NaCl (eqn (5) and (6)). To visualize the induced changes as a function of salt concentration, we calculated the differences of Δ*α*_wrap_ and 
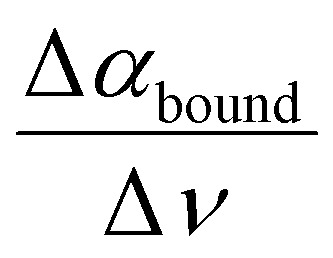
 and Δ*S*_wrap_ and Δ*H*_bound_ referenced to the start conditions without any cosolute (eqn (7)–(10)) in Fig. 2B and C.

7 ΔΔ*α*_wrap_(cosolute) = Δ*α*_wrap_(cosolute) − Δ*α*_wrap_(no cosolute)

8 ΔΔ*S*_wrap_(cosolute) = ΔΔ*α*_wrap_(cosolute)Δ*S̄*_wrap_

9
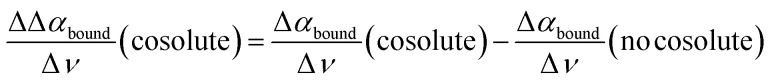
10
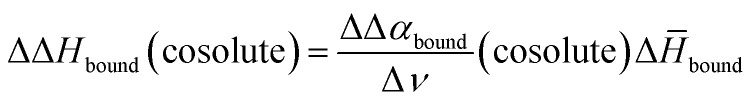


As a result, we find that adding NaCl promotes LLPS by increasing the entropic gain and minimizes the enthalpic loss, see Fig. 2C. Interestingly, in the hydration entropy/enthalpy in Fig. 2C, this is visualized *via* a diagonal which connects measurement points at increasing NaCl concentrations. While this result is expected for cosolutes that promote LLPS, the present result is the first experimental proof that the formation is a consequence of the increase in the released water around hydrophobic groups/patches while the hydration water around hydrophobic groups is retained or even increased upon the addition of NaCl.^65,66^

**Fig. 3:**
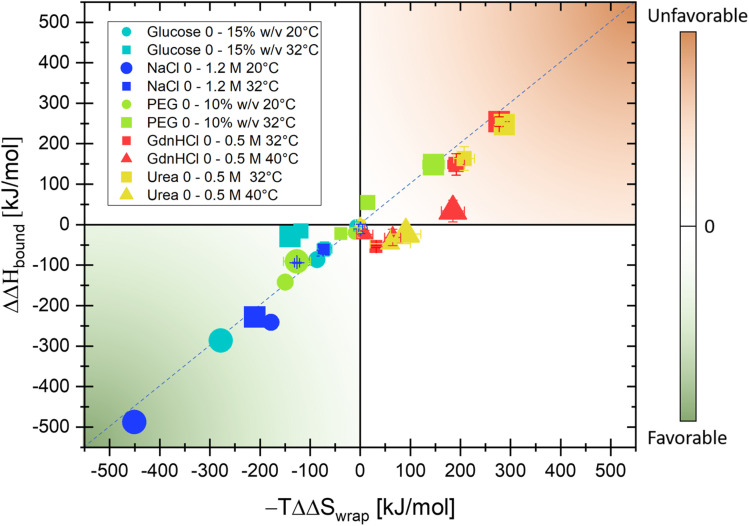
The addition of a co-solute can favor (green quadrant) or disfavor (orange quadrant) liquid–liquid phase separation. The plot summarizes the result of THz calorimetry for changes with respect to hydration entropy (from hydrophobic hydration), plotted on the *x*-axis, and hydration enthalpy (from hydrophilic hydration), plotted on the *y*-axis, upon LLPS when adding co-solutes. The symbols and colors represent the choice of cosolute and their variation with respect to concentration and temperatures, see the legend.

In the following, the same protocol as illustrated in Fig. 2 for NaCl has been applied to several biologically relevant cosolutes (see also turbidity measurements in Fig. S1–S5† and THz spectra in Fig. S6–S10 in the ESI†). Fig. 3 summarizes the results of Δ*S* and Δ*H* upon the addition of cosolutes, PEG, glucose, NaCl, urea, and GdnHCl, at two different temperatures in a hydration entropy/enthalpy plot. Each contribution can be in the order of 100 s of kJ mol^−1^, showing that the addition of cosolutes has a major impact on the hydration free energy, ΔΔ*G* = ΔΔ*H* − *T*ΔΔ*S*, and thereby tunes liquid–liquid phase separation. We want to note that the additional partial entropic and enthalpy solvation contributions upon addition of NaCl, as shown in Fig. 2, will not compensate but will be additive, *i.e.* both will drive LLPS.

We can distinguish between three categories of cosolute that impact LLPs in a specific way by changing the entropic and enthalpic solvation driving forces. Favorable contributions to the free energy upon LLPS are observed for glucose and NaCl at both 20 and 32 °C (green quadrant). By increasing the concentration of each of these cosolutes, favorable changes in both the entropic component due to the release of cavity-wrap water (*i.e.* hydrophobic solvation) and the enthalpic component due to protein–water interactions (bound) are observed. However, at a temperature of 32 °C, the increasing glucose concentration drives LLPS mostly *via* the entropic, hydrophobic solvation term, while the enthalpic component is less impacted (*T* = 32 °C: ΔΔ*H*_bound_ = −29 kJ mol^−1^ and −*T*ΔΔ*S*_wrap_ = −140 kJ mol^−1^ upon addition of 15% w/v glucose).

In contrast, the two co-solutes urea and GdnHCl disfavor or suppress α-elastin's LLPS (orange quadrant). For temperatures of *T* = 32 °C, both partial contributions ΔΔ*S*_wrap_, *i.e.* entropic changes due to hydrophobic hydration, and ΔΔ*H*_bound_, *i.e.* enthalpic changes as deduced by THz calorimetry, are positive (*T* = 32 °C: −*T*ΔΔ*S*_wrap_ = +277 kJ mol^−1^ and ΔΔ*H*_bound_ = +255 kJ mol^−1^, respectively, for 0.5 M of GdnHCl) and therefore disfavor LLPS. At a temperature of 40 °C, this partial entropic contribution is less significant, and the change in hydration enthalpy is almost zero upon addition of GdnHCl (*T* = 40 °C: −*T*ΔΔ*S*_wrap_ = 184 kJ mol^−1^ and ΔΔ*H*_bound_ = 33 kJ mol^−1^ for 0.5 M GdnHCl).

Finally, for PEG we find a bivalent effect, visualized by its presence in both quadrants: at 20 °C the addition of 5% w/v PEG drives LLPS (with a similar mechanism to NaCl and glucose, with ΔΔ*H*_bound_ = −142 kJ mol^−1^ and −*T*ΔΔ*S*_wrap_ = −150 kJ mol^−1^), while at 32 °C the addition of 10% w/v PEG suppresses LLPS (as for GdnHCl and urea, with ΔΔ*H*_bound_ = 158 kJ mol^−1^ and −*T*ΔΔ*S*_wrap_ = 146 kJ mol^−1^).

**Fig. 4:**
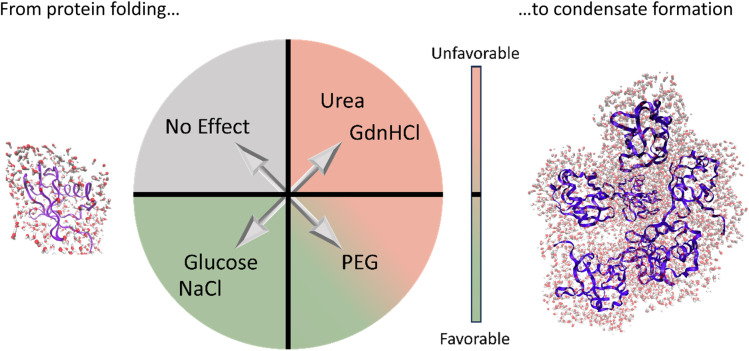
Bridging the cosolute effect from single proteins to protein condensates. The plot summarizes the three classes of cosolutes as found in our THz-calorimetry study, based on their effect on LLPS (*i.e.* on the stability of protein condensates). The classification matches the one known for the cosolute effect on protein stability.^1^ As discussed in the text, a parallelism can be drawn between the two fields, despite the distinct hydration properties of proteins and condensates.

In Fig. 4, we summarize these results and compare these with the well-known effects of the addition of so-solutes on protein stability.^1,2,5,74,75^ Strikingly, the cosolute classification matches for both biological processes, *i.e.* the impact on protein condensate formation is similar to that for protein stabilization. This is remarkable since the hydration properties of protein condensates are thought to be more heterogeneous and dynamic than those of single proteins.^76–78^

This similarity was at first glance unexpected but can be rationalized as follows.

The thermodynamic driving force for protein folding and assembly has been well characterized before.^79^ If we focus on the lower left panel, we see that NaCl and glucose, which are found here to promote LLPS, were also previously observed to promote protein stabilization. In previous studies,^1,2^ NaCl and glucose were proposed to stabilize protein folding by increasing the heat capacity associated with hydrophobic hydration and the exposure to water contact of the nonpolar surface,^80^ since they preferentially bind water, favoring the dewetting of weakly hydrated – hydrophobic protein surfaces. More specifically, it was proposed that NaCl interacts weakly with proteins and that its major effect on protein stability is to decrease the chemical potential of water in the solution, favoring release of hydration water molecules that are weakly bound to the protein surface into the bulk.^1,2,10–13,81–83^ In terms of the present thermodynamic model, this mechanism translates into an increase in the free energy gain from de-wetting of hydrophobic surfaces, similar to that observed for the release of wrapped water molecules from biomolecular condensates probed with THz-calorimetry. Thus, in a similar way to protein stabilization, NaCl and glucose stabilize the protein condensate *via* negative contributions to −*T*ΔΔ*S* due to hydrophobic solvation. Moreover, our findings suggest that the effect of osmolytes, such as NaCl and glucose, does not depend on specific protein–solute interactions, but is general since they mostly contribute by altering the hydration water driving force. The same conclusion was previously reached for protein stability in ref. 81 and 82.

Moving to the upper right panel of the figure, urea and GdnHCl are well-known denaturants that were proposed in several studies to unfold folded protein structures by directly binding to hydrophobic protein surfaces.^14–18,81,82,84–86^ This binding reduces the area of the hydrophobic patches on protein surfaces accessible to water in the unfolded state. In agreement, we observe in our experiments a reduction in the amplitude of the cavity-wrap hydration water component that is released during LLPS, which corresponds to a reduction in the entropic driving force −*T*ΔΔ*S*.

Last, in the bottom right panel, we can observe that PEG has an ambivalent effect on condensate stabilization. Being one of the classical macromolecular crowding agents, PEG was previously proposed to affect protein stabilization with two competing mechanisms: first, we have to consider volume exclusion effects that favor the folded state. Since PEG binds water stronger than protein hydrophobic patches, the free energy cost to wet these patches, which are not exposed to water in the folded state, increases. This effect is balanced by the second mechanism. Protein–PEG interactions disfavor folding by stabilizing the unfolded state, since the binding of PEG to the unfolded protein reduces the hydrophobic protein surface exposed to water. These competing effects were found to be concentration and temperature dependent.^1,44,83,87–90^

In analogy, we find here that, at low temperatures, the entropic contribution from the release of cavity-wrap water is more favorable upon PEG addition, and LLPS is promoted. This can indeed be ascribed to PEG preferentially binding water and decreasing water chemical potential in the solution, causing an increase in the excluded volume effect.^1,44,83,87–90^ We speculate that at high temperature, PEG preferentially binds to protein surfaces instead of staying hydrated, reducing the amount of water that must be released upon LLPS, in a similar way to for protein folding. Since fewer water molecules are displaced upon LLPS, the signals of both wrap and bound fingerprints are reduced in the spectra when adding PEG. This disfavors LLPS.

Generally, our measurements also reveal a high concentration dependence of the solvation driving forces (as shown in Fig. 2 for NaCl as an example), which highlights the importance of crowding effects in cells, as stressed in previous studies on protein stability.^8,9,28^

## Conclusion

We investigate how cosolutes either stabilize or destabilize protein condensates and protein folding. Our experimental results allow deduction of the partial changes in hydration entropy and enthalpy variations upon liquid liquid phase separation in the absence and presence of cosolutes. The resulting free energy imbalance, now accessible with the novel approach of THz-calorimetry, provides a molecular understanding of how small variations in the cosolute concentration within cells can regulate biological functions. By comparing known cosolute effects on protein folding and denaturation, we could show that the same principles hold for both cases, despite the different scale which we cover from a single protein up to protein condensates. These findings not only allow the underlying mechanism to be rationalized but also allow LLPS to be predicted and tuned by the addition of appropriate cosolutes for both biological and medical applications.

## Author contributions

MH designed the study; BK recorded all data; BK and GS analyzed the experimental data; all authors discussed the results and the conclusions, BK, SP and MH wrote and edited the paper.

## Conflicts of interest

There are no conflicts to declare.

## Supplementary Material

SC-016-D4SC07335E-s001

## Data Availability

The data supporting this article have been included as part of the ESI.†
