# Supplementary material for: Tuning biological processes via co-solutes: from single proteins to protein condensates – the case of α-elastin condensation
Source: Chem Sci. 2025 Feb 24;16(14):5897–906. doi: 10.1039/d4sc07335e (PMC11883817; doi:10.1039/d4sc07335e)
Supplement: SC-016-D4SC07335E-s001 [file SC-016-D4SC07335E-s001.pdf]

Supplementary Information (SI) for Chemical Science.
This journal is © The Royal Society of Chemistry 2025

Supporting Information to

# Tuning biological processes via co-solutes: from single proteins to protein condensates – the case of α-elastin condensation

B. König, S. Pezzotti, G. Schwaab, M. Havenith*

Lehrstuhl für Physikalische Chemie II, Ruhr-Universität Bochum, 44780 Bochum, Germany

* Corresponding author

## S1. Turbidity Measurements

UV-visible absorption was recorded on a CS/3D Chirascan-Plus qCD Spectrometer (Applied Photophysics Ltd. 21, Leatherhead, UK) using a 2 mm micro cuvette (Hellma, Müllheim, Germany). LLPS was qualitatively assayed by monitoring turbidity through light scattering at 400 nm at selected temperatures between 12 and 40 °C. Therefore, a stepped temperature ramp in 4°C steps was performed with a 3 minutes thermalization period at each temperature. The temperature of the sample cell was controlled by the integrated Peltier element with a tolerance of 0.10°C. [1]

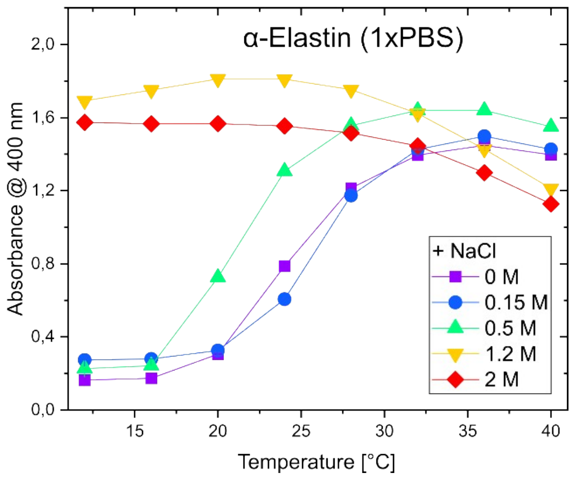


Fig.1: Turbidity measurements at 400 nm for 20 mg/mL α-elastin for different added NaCl concentrations and temperatures. UV/vis absorption spectrum of 20 mg/mL α-elastin 1xPBS buffer solutions at different temperatures (12-40°C).

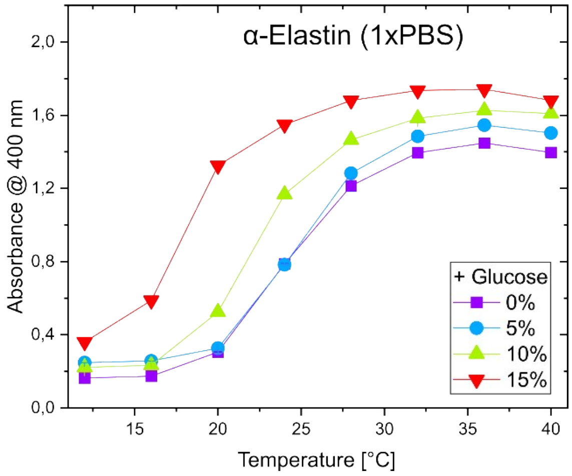


Fig.2: Turbidity measurements at 400 nm for 20 mg/mL α-elastin for different added glucose concentrations and temperatures. UV/vis absorption spectrum of 20 mg/mL α-elastin 1xPBS buffer solutions at different temperatures (12-40°C).

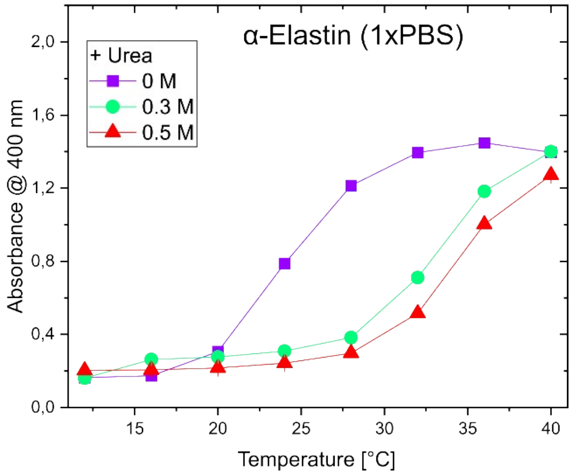


Fig.3: Turbidity measurements at 400 nm for 20 mg/mL α-elastin for different added urea concentrations and temperatures. UV/vis absorption spectrum of 20 mg/mL α-elastin 1xPBS buffer solutions at different temperatures (12-40°C).

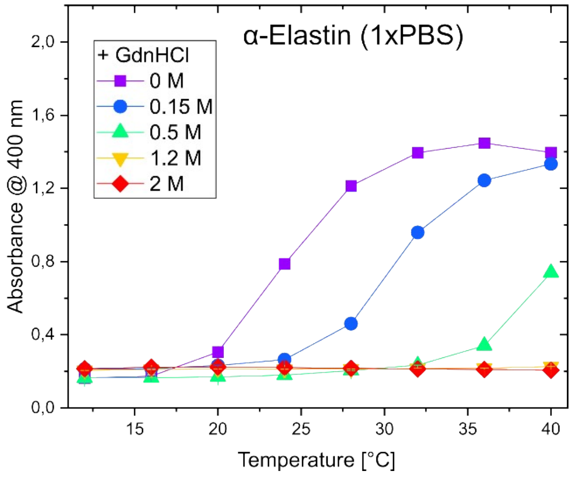


Fig.4: Turbidity measurements at 400 nm for 20 mg/mL α-elastin for different added GdnHCl concentrations and temperatures. UV/vis absorption spectrum of 20 mg/mL α-elastin 1xPBS buffer solutions at different temperatures (12-40°C).

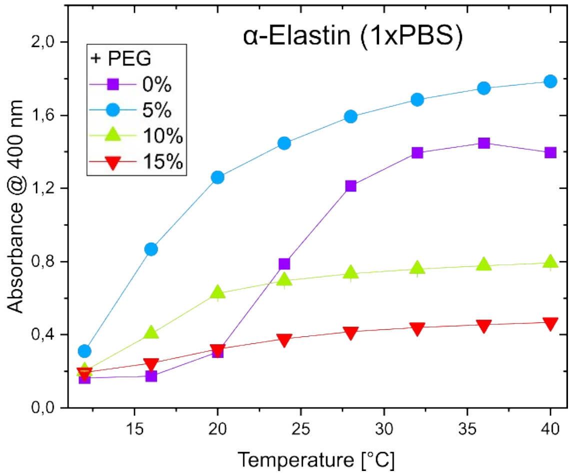


Fig.5: Turbidity measurements at 400 nm for 20 mg/mL α-elastin for different added PEG concentrations and temperatures. UV/vis absorption spectrum of 20 mg/mL α-elastin 1xPBS buffer solutions at different temperatures (12-40°C).

## S2. THz Difference Spectra

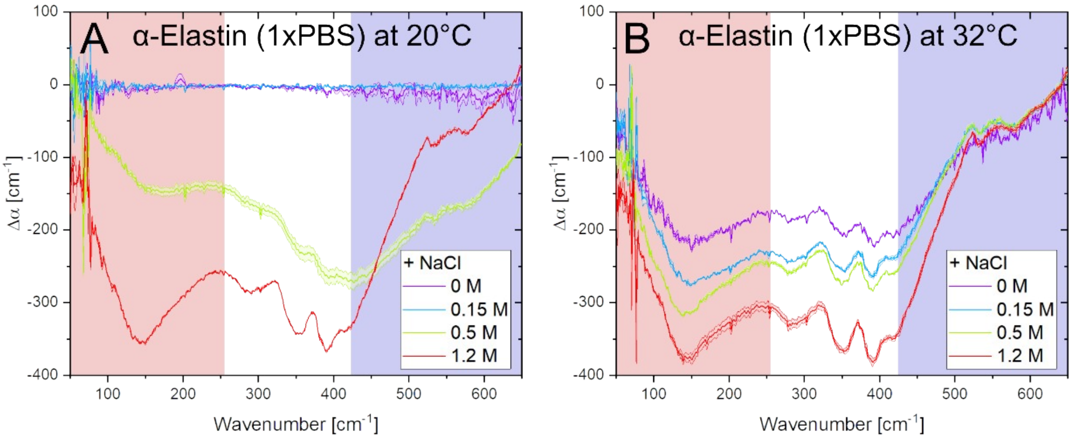


Fig.6: Δα spectra reveal the stabilization of α-elastin's LLPS upon increasing NaCl concentrations at two temperatures. Δα spectra upon saturation point LLPS of 20 mg/mL α-elastin (1xPBS) upon the increase of NaCl concentration (0, 0.15, 0.5 and 1.2 M) at 20°C (A) and 32°C (B). With higher NaCl concentration LLPS can be triggered already at 20°C. This stabilization is also visible at 32°C with increasing amplitudes with increasing NaCl concentration.

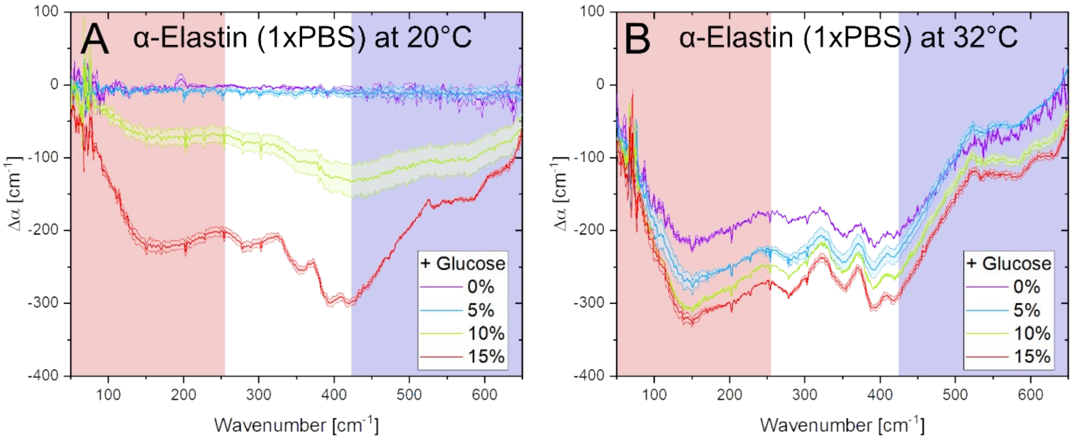


Fig.7: Δα spectra reveal the stabilization of α-elastin's LLPS upon increasing glucose concentrations at two temperatures. Δα spectra upon saturation point LLPS of 20 mg/mL α-elastin (1xPBS) upon the increase of glucose concentration (0,5,10 and 15% w/v) at 20°C (A) and 32°C (B). With higher glucose concentration LLPS can be triggered already at 20°C. This stabilization is also visible at 32°C with increasing amplitudes with increasing glucose concentration.

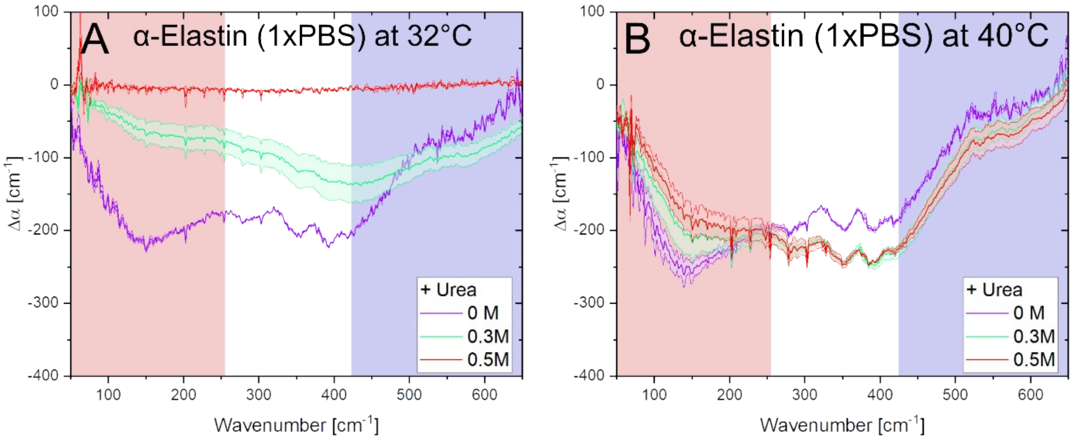


Fig.8: Δα spectra reveal the destabilization of α-elastin's LLPS upon increasing urea concentrations at two temperatures. Δα spectra upon saturation point LLPS of 20 mg/mL α-elastin (1xPBS) upon the increase of urea concentration (0, 0.3, 0.5 M) at 32°C (A) and 40°C (B). With higher urea concentrations LLPS can be suppressed at 32°C. This destabilization is also visible at 40°C with decreasing low frequency amplitudes with increasing urea concentration, while the higher frequency is less affected.

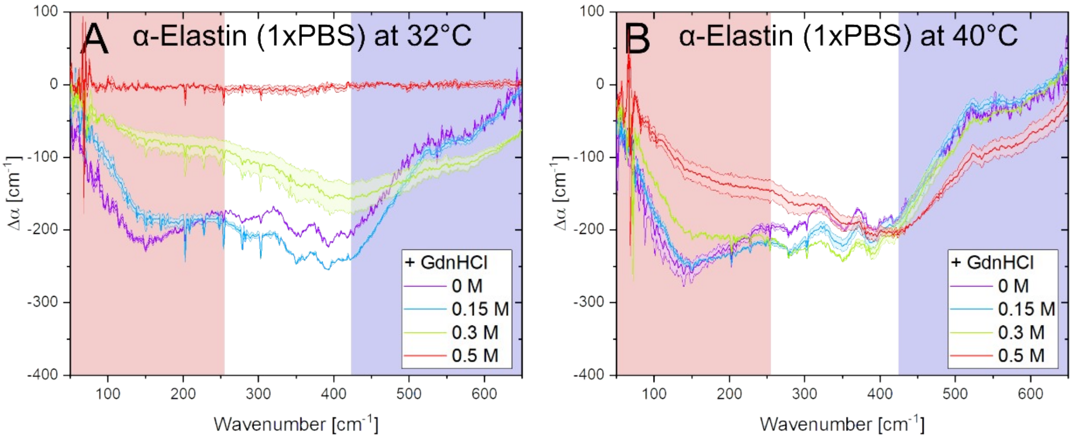

Fig.9: Δα spectra reveal the destabilization of α-elastin's LLPS upon increasing GdnHCl concentrations at two temperatures. Δα spectra upon saturation point LLPS of 20 mg/mL α-elastin (1xPBS) upon the increase of GdnHCl concentration (0, 0.15, 0.3, 0.5 M) at 32°C (A) and 40°C (B). With higher GdnHCl concentrations LLPS can be suppressed at 32°C. This destabilization is also visible at 40°C with decreasing low frequency amplitudes with increasing GdnHCl concentration, while the higher frequency is less affected.

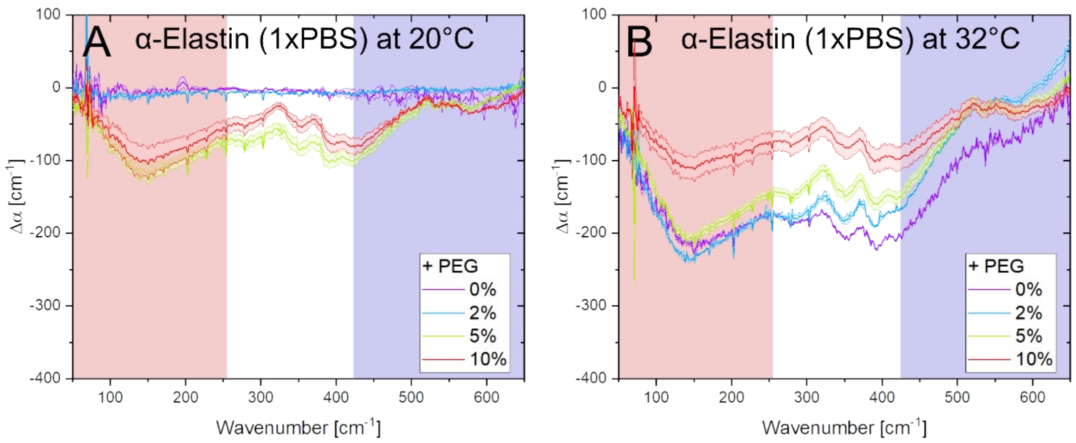


Fig.10: Δα spectra reveal the changing influence of α-elastin's LLPS upon increasing PEG concentrations at two temperatures. Δα spectra upon saturation point LLPS of 20 mg/mL α-elastin (1xPBS) upon the increase of PEG concentration (0, 2, 5 and 10% w/v) at 20°C (A) and 32°C (B). With higher PEG concentration LLPS is initially stabilized, while it is reaching a turning point at high concentrations starts to destabilize the process. This turning point is reached earlier at 32°C.

## References

[1] J. Möller, S. Grobelny, J. Schulze, S. Bieder, A. Steffen, M. Erlkamp, M. Paulus, M. Tolan, R. Winter, *Phys. Rev. Lett.* **2014**, *112*, 1–5.
